# California Serogroup Virus Infection Associated with Encephalitis and Cognitive Decline, Canada, 2015

**DOI:** 10.3201/eid2308.170239

**Published:** 2017-08

**Authors:** Duncan Webster, Kristina Dimitrova, Kimberly Holloway, Kai Makowski, David Safronetz, Michael A. Drebot

**Affiliations:** Dalhousie University, Saint John, New Brunswick, Canada (D. Webster);; Public Health Agency of Canada, Winnipeg, Manitoba, Canada (K. Dimitrova, K. Holloway, K. Makowski, D. Safronetz, M.A. Drebot)

**Keywords:** Bunyaviridae, Orthobunyavirus, California serogroup virus, arbovirus, meningitis/encephalitis, viruses, New Brunswick, Canada, Manitoba, Canada, mosquito-borne, febrile illness, neurologic disease, Jamestown Canyon virus, snowshoe hare virus, cognitive impairment, cognitive decline, vector-borne infections

## Abstract

California serogroup (CSG) viruses, such as Jamestown Canyon and snowshoe hare viruses, are mosquitoborne pathogens that cause febrile illness and neurologic disease. Human exposures have been described across Canada, but infections are likely underdiagnosed. We describe a case of neuroinvasive illness in a New Brunswick, Canada, patient infected with a CSG virus.

California serogroup (CSG) viruses (family *Bunyaviridae*, genus *Orthobunyavirus*) (*1*) include the mosquitoborne pathogens Jamestown Canyon virus (JCV), snowshoe hare virus (SSHV), and La Crosse virus. Competent vectors include non-*Culex* mosquitoes (e.g., *Aedes*, *Culiseta*, and *Anopheles* species), all of which circulate in New Brunswick, Canada (*2*,*3*). The major reservoir and vertebrate amplifier of JCV is believed to be the white-tailed deer (*4*). Squirrels, chipmunks, and hares serve as vertebrate reservoirs for SSHV (*5*). CSG virus infection is generally asymptomatic; however, after an incubation period of 3–7 days, a febrile illness may develop, and central nervous system involvement may lead to encephalitis or meningoencephalitis (*6*). No targeted therapies exist; treatment is supportive.

We describe a previously independent 73-year-old man, living on Grand Manan Island, off the Fundy Coast of southern New Brunswick, with a febrile illness that began July 23, 2015. The man was hospitalized, and the next day he exhibited abnormal behaviors (purposeless movements and incoherent speech) and became increasingly confused. The confusion and fever continued to worsen, and headache and neck pain developed. On July 28, he was treated with ceftriaxone and transferred to a tertiary-care hospital (Saint John Regional Hospital, Saint John, New Brunswick), where ampicillin and acyclovir were administered. Cerebral spinal fluid values were as follows: leukocyte count, 1 × 10^6^ cells/L (reference range 0–5 × 10^6^ cells/L); glucose, 3.5 mmol/L (reference range 2.2–3.9 mmol/L); and protein concentration, 0.58 g/L (reference range 0.15–0.45 g/L). On July 30, an infectious diseases specialist diagnosed the patient with viral encephalitis, most likely secondary to herpes simplex virus infection; ceftriaxone and ampicillin were discontinued. At a neurology consultation on July 31, the patient was still febrile and confused; brain magnetic resonance imaging revealed no acute pathology.

On August 4, additional information revealed that the patient went on frequent excursions into the woods of Grand Manan Island and had exposures to feral cats. Doxycycline was initiated, and serologic tests were conducted for *Bartonella*, *Borrelia*, *Coxiella*, and *Anaplasma* species and for Powassan virus, JCV, and SSHV; tests were also conducted to rule out paraneoplastic process and autoimmune causes. By August 12, the patient was afebrile but remained confused. On August 18, PCR was negative for herpes simplex virus in CSF, and an electroencephalograph revealed no periodic lateralizing epileptiform discharges; acyclovir was discontinued. Initial serologic test results were negative, and the patient showed no autoimmune or paraneoplastic markers. During a geriatric assessment, the patient showed persistent delirium, scoring 13/30 on a mini–mental state examination.

On August 21, we received the patient’s initial CSG serology results and collected follow-up serum samples for standard serologic testing by IgM capture ELISA and plaque-reduction neutralization testing (PRNT) (*7*,*8*). The acute-phase serum sample was IgM-negative but positive for CSG virus–specific neutralizing antibodies by PRNT (Table). Serial convalescent-phase serum specimens demonstrated IgM seroconversion several weeks after symptom onset and a >4-fold rise in PRNT titers for JCV and SSHV, indicating a diagnostic rise. Because serum neutralization titers for both viruses were equivalent or, at most, demonstrated only a 2-fold difference, the specific CSG virus associated with the patient’s illness could not be determined. Confirmatory PRNT titers for both viruses and the absence of IgM in the acute-phase serum suggest prior exposure to a CSG virus associated with the etiologic pathogen in this case. Secondary infections with orthobunyaviruses may result in a gradual or delayed rise in IgM, with neutralizing antibodies already detectable early after symptom onset, as documented in other cases involving CSG viruses (*9*).

**Table:** Serologic test results for a patient with California serogroup virus infection associated with encephalitis and cognitive decline, Canada, 2015*

Virus	July 31	August 10	August 21
Jamestown Canyon virus			
ELISA IgM	Negative	Equivocal	Positive
PRNT titer	1:40	1:160	1:320
Snowshoe hare virus			
ELISA IgM	Negative	Equivocal	Positive
PRNT titer	1:20	1:320	1:320

*PRNT, plaque reduction neutralization test.

Results for all other serologic tests were negative, leading to a modification of the diagnosis to confirmed CSG viral encephalitis. On September 9, the patient was transferred to a nursing home. In January 2016, further assessment revealed a lower mini–mental state examination score of 11/30. The patient, who was totally dependent for personal care and instrumental activities of daily living, was diagnosed with postencephalitic dementia.

Exposures to CSG viruses have been documented in New Brunswick (*5*; M.A. Drebot, unpub. data), and serologic results described in this report suggest that the patient may have been infected by JCV or SSHV on 2 different occasions. Human seroprevalence of CSG virus, specifically JCV, has been noted to be high in the maritime provinces of Atlantic Canada. A serosurvey in Nova Scotia identified an overall CSG seroprevalence of 21.2% (95% CI 16.1%–27.0%) (*10*). As such, there is need for increased awareness that these viruses are circulating during the mosquito season and may be associated with human disease.

Although most CSG infections result in mild illness, this case further highlights that these viruses can cause severe and debilitating neuroinvasive disease. Patients who seek medical care for febrile or encephalitic clinical symptoms and who have possible or known exposures to mosquito vectors should be considered for CSG virus testing. JCV and SSHV infection should be considered in the differential diagnosis for such patients during the spring, summer, and fall.
